# Network-based validation of the psychometric questionnaire EDI-3 for the assessment of eating disorders

**DOI:** 10.1038/s41598-023-28743-5

**Published:** 2023-01-28

**Authors:** Clara Punzi, Paolo Tieri, Laura Girelli, Manuela Petti

**Affiliations:** 1 Data Science Program, Sapienza University of Rome, Via Ariosto 25, 00185 Rome, Italy; 2 CNR National Research Council, IAC Institute for Applied Computing, Via dei Taurini 19, 00185 Rome, Italy; 3 Department of Humanities, Philosophy and Education, University of Salerno, via Giovanni Paolo II 132, 84084 Fisciano, Italy; 4 Department of Computer, Control, and Management Engineering “Antonio Ruberti”, Sapienza University of Rome, Via Ariosto 25, 00185 Rome, Italy

**Keywords:** Psychology, Human behaviour, Quality of life

## Abstract

Assessing the validity of a psychometric test is fundamental to ensure a reliable interpretation of its outcomes. Few attempts have been made recently to complement classical approaches (e.g., factor models) with a novel technique based on network analysis. The objective of the current study is to carry out a network-based validation of the Eating Disorder Inventory 3 (EDI-3), a questionnaire designed for the assessment of eating disorders. Exploiting a reliable, open source sample of 1206 patients diagnosed with an eating disorder, we set up a robust validation process encompassing detection and handling of redundant EDI-3 items, estimation of the cross-sample psychometric network, resampling bootstrap procedure and computation of the median network of the replica samples. We then employed a community detection algorithm to identify the topological clusters, evaluated their coherence with the EDI-3 subscales and replicated the full validation analysis on the subpopulations corresponding to patients diagnosed with either anorexia nervosa or bulimia nervosa. Results of the network-based analysis, and particularly the topological community structures, provided support for almost all the composite scores of the EDI-3 and for 2 single subscales: Bulimia and Maturity Fear. A moderate instability of some dimensions led to the identification of a few multidimensional items that should be better located in the intersection of multiple psychological scales. We also found that, besides symptoms typically attributed to eating disorders, such as drive for thinness, also non-specific symptoms like low self-esteem and interoceptive deficits play a central role in both the cross-sample and the diagnosis-specific networks. Our work adds insights into the complex and multidimensional structure of EDI-3 by providing support to its network-based validity on both mixed and diagnosis-specific samples. Moreover, we replicated previous results that reinforce the transdiagnostic theory of eating disorders.

## Introduction

Eating Disorders (EDs) are characterized by severe disturbances in people's eating or eating-related behavior, emotions, and weight-related thoughts (e.g., preoccupation with food, body weight and shape). The typical age at onset is in early to late adolescence^1,2^, however the onset can occur virtually throughout the lifespan^3^. EDs can be classified as anorexia nervosa (AN), bulimia nervosa (BN), binge-eating disorder (BED), and other specified feeding and eating disorders (OSFED)^4^. EDs are traditionally considered more frequent in women and several studies have confirmed this prevalence rate^5^. However, this finding may vary according to the type of ED. Males are usually considered to account for 10% of subjects with AN or BN^4^. However, other studies indicate that ≤ 25% of subjects with AN or BN are males and that males account for 36% of subjects with all typical BEDs^6^. Furthermore, studies conducted in non-clinical settings have shown that these gender differences are likely to be more nuanced in community-based samples^7^ and this may probably be due to biases in reporting^8,9^.

It is to note that the theoretical and clinical framework under which EDs are approached has been built based on the experience of a vast majority of female patients. Consequently, If there existed aspects of the psychopathology exclusive of men, then it is likely that they would not be measured. Hence, as Perko^9^ pointed out, the seriousness and diffusion of EDs in men could be actually masked by such an incomplete framework. Mortality rates for AN and BN are the highest in psychiatric conditions and higher than many medical conditions^10^, therefore early recognition and treatment of these disorders are fundamental to avoiding a chronic course.

Although the accurate detection of symptoms, behaviors, physical signs, syndrome combinations, and duration by a trained clinician is essential for a correct diagnosis, some instruments have been designed in support of clinical experience for the recognition of the clinical signs of eating disorders. The Eating Disorder Inventory 3 (EDI-3^11–13^) is among the tools commonly used to evaluate EDs^14^. The EDI-3 is a standardized, self-report measure that has the advantage of assessing eating disorder symptoms and associated psychological characteristics, in contrast to other measures that are limited to assessing eating disorder symptoms^15,16^. The questionnaire has also been largely used by researchers for assessing areas of the psychopathology of interest in theory-testing and assessing treatment outcomes^17^.

The scale consists of 91 items organized into 12 primary scales, consisting of 3 eating-disorder-specific scales and 9 general psychological scales^12^. Several studies have analyzed the psychometric properties of the scale^18,19^ and their validity through latent factor models^12,13,20,21^. Despite generally supporting the validity and the twelve theoretical dimensions of the scale, the findings of these studies still leave some questions open about which of these models fits the data better: a second-order factor model—twelve primary latent factors + two second order factors (eating disorder risk and psychological disturbance)^12,20^, a bifactor model—twelve primary latent factors allowed to correlate + a bifactor (general distress) orthogonal to the twelve primary latent factors, with all the items loading on it^13,21^, or an Exploratory Structural Equation Modeling (ESEM) two-bifactor model—twelve primary latent factors allowed to correlate + two bifactor, orthogonal rotation^21^.

Furthermore, over the past decade, critiques of the application of latent factor models to psychopathology have been largely increasing^22–25^. Latent factor models assume the property of local independence among symptoms, which are considered manifestations of some common underlying factor and passive receptors of its causal influence. However, not only this assumption cannot be proved true in most psychiatric disorders, including EDs, but it is not even confirmed by the DSM criteria, which often report causal relations between symptoms of the same disorders^23,26^.

In the attempt to overcome this pitfall of the latent factor model, a new conceptualization of mental disorders from a network perspective emerged in the last decades. According to this view, symptoms are seen as mutually interacting and reciprocally reinforcing elements that do not passively arise as the consequence of a specific underlying mental disorder, but instead constitute their causally active components^22–26^. Remarkably, recent work brought new evidence of the fact that the latent factor and network approaches do not actually exclude each other but should rather be integrated in the so called “generalized network models”. Indeed, particular hybrid models have been proven to have a better fit compared to network-only models^27,28^.

Recently, the new conceptualizations of both network-only and generalized network models have also been applied to EDs as a promising approach to provide a clearer understanding of such complex disorders and inform future prevention and treatment strategies^29^ (for a comprehensive appraisal see^30,31^). However, despite the exponentially increasing number of studies employing the network approach to analyze this psychopathology, and test its benefits in clinical intervention^32–34^ to our knowledge no study so far has applied the network model to examine the validity of measures assessing mental disorders. This is an important gap in the literature as the selection of an adequate model should rely on congruence between methodology and theory^35^.

Little recent work has analyzed the validity of psychological measures through network analysis. Differently from these studies, which focused on personality traits, well-being, and positive and negative affects^36–38^, the present study aims to analyze for the first time the validity of a questionnaire designed for the assessment of a class of mental disorders, specifically of eating disorders, from a network perspective.

The concept of validity of a psychometric questionnaire is not univocally defined. Within the latent variable framework, Borsboom^39^ introduced the conception of “test validity” stating that this is a property of tests such that a given questionnaire can be said to be valid for measuring an attribute if and only if (a) the attribute exists (i.e., the attribute exists prior to and independently of the questionnaire), and (b) variations in the attribute causally produce variations in the test scores. Although simple and effective, this definition has its own limitations, such as its applicability to formative models where, in contrast to reflective models, the indicators encoded in the test are not interchangeable because each item contributes a specific meaning to the latent variable.

From the network perspective, Christensen^37^ claimed that psychometric questionnaires measure the state of the network composed of causal connected components (i.e. an item or set of items that share a unique causal system and are causally independent from other components). Notably, this definition implies that the domain of a psychological trait or attribute might be given by the combination of multiple components, that is, heterogeneous causal processes. Hence, one should assess the validity of a test with respect to such components and not to single attributes as in the case of the factor model perspective.

The expression “validation” refers instead to the process of estimating the extent to which empirical data and theoretical justifications support the validity of the questionnaire^39^. Although such a process can be differently approached, by “validation” we specifically refer to “structural validation”, that is, a range of quantitative analyses aimed at examining the psychometric properties of a test (e.g., its factor structure or internal consistency). The methodological steps applied during the validation process clearly depend on the postulated definition of validity. Actually, the network-based validation of a psychometric measure does not oppose but rather complements the classical factor-model validation by adding important insights on the properties of the test, such as the relationship between items or groups of items^37^, and, in particular, its community structure that might uncover the existence of groups of internally highly correlated items.

In the following, we will often mention the “diagnosis-specific validation” of the psychometric test in reference to a particular characterization of the subsample employed for validation. More precisely, given the heterogeneous composition of our sample in terms of ED diagnosis, we run and compared the validation of the questionnaire based on both the full sample (i.e., with mixed diagnosis), and the subsamples of patients diagnosed with a specific ED (i.e., diagnosis-specific). Such differentiated validation was not appraised in one of the most recent assessments of the EDI-3 structural properties via factor models^13^ as he claimed that the considerable within-group variance on the EDI-3 scales supports the relative stability of the traits measured by EDI-3 and thus would result in poor interpretative value. However, even if that was the case within the framework of factor models, the same might not hold true when moving to the network-based perspective we are discussing in this paper. Indeed, important suggestions about different relationships of cross-domain items could emerge while investigating and comparing networks corresponding to distinct diagnostic groups. Two questions arise from this observation: the first is whether EDI-3 is able to retrieve such causal interaction independently of the diagnostic group, and second, how can we interpret the results of dimension analysis whenever they differ depending on the sample composition.

This paper is composed of two major sections, the first aimed at validating the EDI-3 questionnaire from a network perspective (research questions RQ1 and RQ2 below); the second aimed at assessing its diagnosis-specific validity, as well as at identifying and comparing the core symptoms or psychological traits of both the mixed and diagnosis-specific networks to highlight similarities and plausible differences that might be targeted by clinicians to disrupt the network and prevent further activation of symptoms^24,40^ (research questions RQ3 and RQ4 below).

More specifically, we organized our work to answer the following research questions:

**Section 1: Structural validation of EDI-3**

1. Is the EDI-3 structurally valid from a network perspective? That is, is there any evidence that it can give a valid evaluation of its causally connected components (i.e., psychological dimensions)?
2. How can the structural validity estimated with the tools of network analysis complement the results of the latent variable model validation of EDI-3?

**Section 2: Diagnosis-specific validation of EDI-3**

RQ3. Within each diagnostic group, a remarkable variability on EDI-3 psychological scales is expected^13^. What are the implications of this situation on the validity of the questionnaire? That is, can the EDI-3 be considered valid from the network perspective when administered to specific diagnostic groups?
RQ4. Does our data support the transdiagnostic theory of EDs^41^? To what extent do the central nodes of the symptom network built from the cross-sample dataset of patients (i.e., with mixed diagnosis of EDs) differ from those of the symptom networks corresponding to each diagnostic group?

## Materials and methods

### Participants and procedures

**Figure 1:**
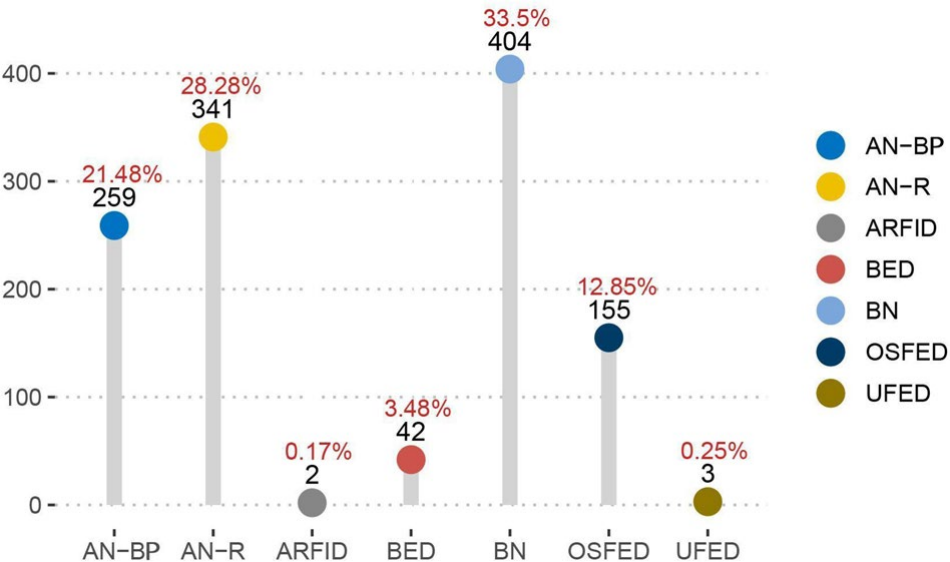
Frequency of different DSM-5 diagnoses for EDs in the sample collected by Brookings (2020)^15^.

We used data from the open access project Eating Disorder Inventory-3, American clinical cases^15^ available on the openICPSR repository (https://www.openicpsr.org/openicpsr/project/109443/version/V2/view;jsessionid=21FD8DB3130A73F094AEA1D5AFD21874). The dataset consists of a large sample of 1206 female patients that were admitted to an eating disorder clinic in Ohio between 1996 and 2015. They were all North American residents with ages in the range 11–75 (μ = 22.58, σ = 8.88). At the time of data collection, all participants met the DSM-5 criteria for the diagnosis of an eating disorder (ED), with diagnostic rates as illustrated in Fig. 1 and Table 1. The raw dataset did not contain any missing values, noting that the expectation–maximization algorithm was used to impute data in 407 cases where one to five responses were missing^13^.

**Table 1:** Demographic characteristics of patients, grouped by ED diagnoses. All patients were female.

Diagnosis	Age	Total n (%)
AN^a^	11–63 (μ = 22.6, σ = 9.62)	600 (49.8)
BN	13–52 (μ = 22.11, σ = 6.94)	404 (33.5)
Others^b^	11–75 (μ = 23.44, σ = 9.94)	202 (16.7)
Total	11–75 (μ = 22.58, σ = 8.88)	1206 (100)

AN, Anorexia Nervosa; AN-R, AN-Restrictive type; AN-BP, AN-Binge Purging type; BN, Bulimia Nervosa; BED, Binge Eating Disorder; ARFID, Avoidant Restrictive Food Intake Disorder; OSFED, Other Specified Feeding or Eating Disorder; UFED, Unspecified Feeding or Eating Disorder.

^a^AN = AN-R + AN-BP.

^b^Others = BED + ARFID + OSFED + UFED.

### Measures

Eating disorders symptoms and related psychological traits were assessed with the Eating Disorder Inventory-3^12^ (EDI-3). EDI-3 is a self-report questionnaire consisting of 91 items rated on a five-point (0–4) Likert scale and organized into 12 primary subscales consisting of 3 EDs specific scales, namely drive for thinness (DT), bulimia (B), and body dissatisfaction (BD), and 9 general psychological scales that are highly relevant to, but not specific to, eating disorders, namely low self-esteem (LSE), personal alienation (PA), interpersonal insecurity (II), interpersonal alienation (IA), interoceptive deficits (ID), emotional dysregulation (ED), perfectionism (P), asceticism (A), and maturity fears (MF). EDI-3 yields six composite scores: one that is eating-disorder specific (i.e., eating disorder risk) and five that are general integrative psychological constructs (i.e., ineffectiveness, interpersonal problems, affective problems, overcontrol, general psychological maladjustment). The complete questionnaire is available at^12^.

### Structural validation of EDI-3

Similarly to previous works^36–38^, the structural validation of EDI-3 was carried out through the following major stages: the identification and handling of possible redundant nodes (redundancy analysis); network estimation; the detection of topological communities within the network (dimension analysis); and, finally, the evaluation of the extent to which items in each dimension are homogeneous (i.e., they are causally coupled) and interrelated given the questionnaire’s multidimensional composition.

The full process of data analysis was managed with the statistical environment R (Version 4.1.2) through the open-source application RStudio (2021.09.1.372). Many existing R packages are specifically designed for the psychometric network estimation and analysis, in particular EGAnet^42^, qgraph^43,44^, bootnet^45,46^, networktools^47^, and igraph^48^ (for a comprehensive review see^31,49^).

#### Redundancy analysis and network estimation

Within the network approach, the first step of scale validation is to identify and handle redundant nodes. In fact, a fundamental tenet of network psychometrics is that a psychological construct (i.e. personality, well-being) or a mental disorder (depression, EDs) is made up of unique causal components (i.e. symptoms of a mental disorder) which thus cannot be exchanged with other components of the system^50–52^. Therefore, to reduce latent confounding and do not mine the interpretability of nodal metrics, these components should be unique rather than redundant^53^. Since most existing psychological scales have been developed from a latent variable perspective, they are likely to have items that are not unique^54^. Consider, for example, the items *“I binge eat”* and *“I think about binging”*, they clearly have a common underlying attribute: binge eating. From the psychometric network perspective, these items are not unique components themselves but comprise a single unique component. This makes the identification of redundant nodes in the network a priority for the EDI-3 validation from a network perspective.

In the current study, we checked for the presence of redundant nodes in the raw dataset composed of 1206 individuals and 91 variables employing the Unique Variable Analysis (UVA)^55^ implemented in the EGAnet R package. We decided in favor of this method since it provides a detailed graphical interface for the visualization of redundancies between two or more variables and it also allows for a tailored selection of which redundant variables to combine. To assess node similarities and their related significance level, we employed the weighted topological overlap and the adaptive alpha, respectively^56^. More precisely, node pairs with topological overlap greater than zero were firstly selected to compute the probability (i.e., *p*-values) of achieving their corresponding value from a fitted distribution. Next, a multiple comparison method was used to identify the redundant node pairs as those whose p-values were less than the corrected alpha. Importantly, since items that are redundant with a target item may also be redundant with other items that are not redundant with the target, “redundancy chains” could also emerge, suggesting the combination of more than two items at once. To handle this situation, we chose to employ the heuristic of combining only those variables that were either directly connected to the target or that were involved in cliques, since these have been suggested to be indicators of highly overlapping items^37,57^. In both cases, the reduction step was carried out by fitting a confirmatory factor analysis model. Apart from UVA, other methods exist to handle redundancy in questionnaires. In particular, we assessed the extent to which the UVA method proposed and implemented by Christensen^37^ differs from other methods typically used in other fields (e.g., psychometric network analysis) to assess item redundancies (see Supplementary material 1, section 2).

Once the redundancy-corrected set of items (EDs symptoms and personality traits) was identified, we estimated the psychometric network as a regularized partial correlation network through a Gaussian Graphical Model with LASSO regularization (shortly, GLASSO^58^).

#### Dimension analysis: evaluating the agreement between topological communities and EDI-3 subscales

Dimensionality assessment is an integral step for validating the structure of a questionnaire. In psychometric networks, this analysis is performed using community detection algorithms. These algorithms identify densely connected sets of nodes (communities) that form coherent subnetworks within the overall network: these subnetworks represent the dimensions of the scale under investigation.

In this study, we used the Spinglass algorithm implemented in the igraph R package to detect communities^48,59^: differently from other methods, it can handle negative edge weights and it is considered a good choice in the case of small networks such as the one under consideration here^60^. Additionally, we explored the coherence of the detected dimensions by inspecting the loading matrix, that is, a matrix reporting the standardized node strength for each node in each dimension. Although it might be interpreted similarly to a traditional factor loading matrix (indeed, their equivalence has been proved in^61,62^), the network loadings differ quantitatively in that the values correspond to partial correlations instead of zero-order correlations. Consequently, their magnitude is much lower, in particular, that of cross-loadings, whose value is furtherly decreased by the shrinkage operation applied in the network estimation step, where the $L1$-norm LASSO penalty shrinks the regression coefficients towards zero with the aim of determining a sparse inverse covariance matrix.

Importantly, in order to avoid any bias in the network estimation due to our specific dataset, we employed a resampling bootstrap procedure (500 replications of Bootstrap Exploratory Graph Analysis^42,63^) and finally estimated a median network structure with nodes corresponding to the reduced EDI-3 items and edges computed as the median of all pairwise correlations over all replica samples. Given this median network, we compared its topological community structure with the EDI-3 subscales to estimate the extent of their agreement. Furthermore, we identified possible nodes causing low stability and tried to understand the reasons.

We also analyzed the empirical network (i.e., the GLASSO network estimated from the raw dataset) in comparison to the median network. In particular, we assessed both its structural consistency and item stability to completion of internal consistency (see Supplementary material 1, section 3).

### Diagnosis-specific validation of EDI-3

In the attempt to answer the questions about possible fluctuations in the interactions between dimensions across diagnostic groups, we further inspected the validity of EDI-3 on specific subpopulations, namely those composed of patients diagnosed with two of the three typical EDs: anorexia nervosa, either the restrictive (AN-R) or binge-purging (AN-BP) subtype, and bulimia nervosa (BN). The selection of such specific groups was essentially dictated by statistical reasons: firstly, they were the most represented diagnoses in our dataset, and thus ensured a more reliable network estimation; secondly, the difference in their cardinality was not that marked to affect the quality of their comparison.

We replicated the redundancy and dimension analyses as for the cross-sample network and we examined the coherence between the detected topological dimensions not only with the psychological subscales, but also with the results of the cross-sample network. In particular, we compute the mutual information score to assess the agreement of the community structures determined on the 91-variable graph by each of three populations studied in the current paper: the cross-sample, the AN and BN subsamples.

To complete our analysis, we investigated the strength centrality index, a well established nodal metric in psychometric network analysis^26,31,49^, of all items in the cross-sample, AN and BN symptom networks with the aim of highlighting plausible discrepancies that might help clinicians discriminate between different diagnostic groups. Compared to other metrics assessing nodal influence (e.g., expected influence^64^, strength centrality resulted to be the one characterized by the highest stability across all the datasets analyzed in the current study (see Supplementary Material 1, section 4).

## Results

### Structural validation of EDI-3

#### Redundancy analysis

As a result of the redundancy analysis, the number of variables in the systems decreased from 91 to 58. Since UVA detected many redundancy chains, we defined several new variables as the combination of a number of items ranging from 2 to 5. The list of merged nodes is reported in Table S1. As expected, the items in all redundancy chains were part of the same subscale. Notably, no redundancy was detected within the PA and IA subscale, while just one pair was identified within A and LSE.

#### Dimension analysis

**Figure 2:**
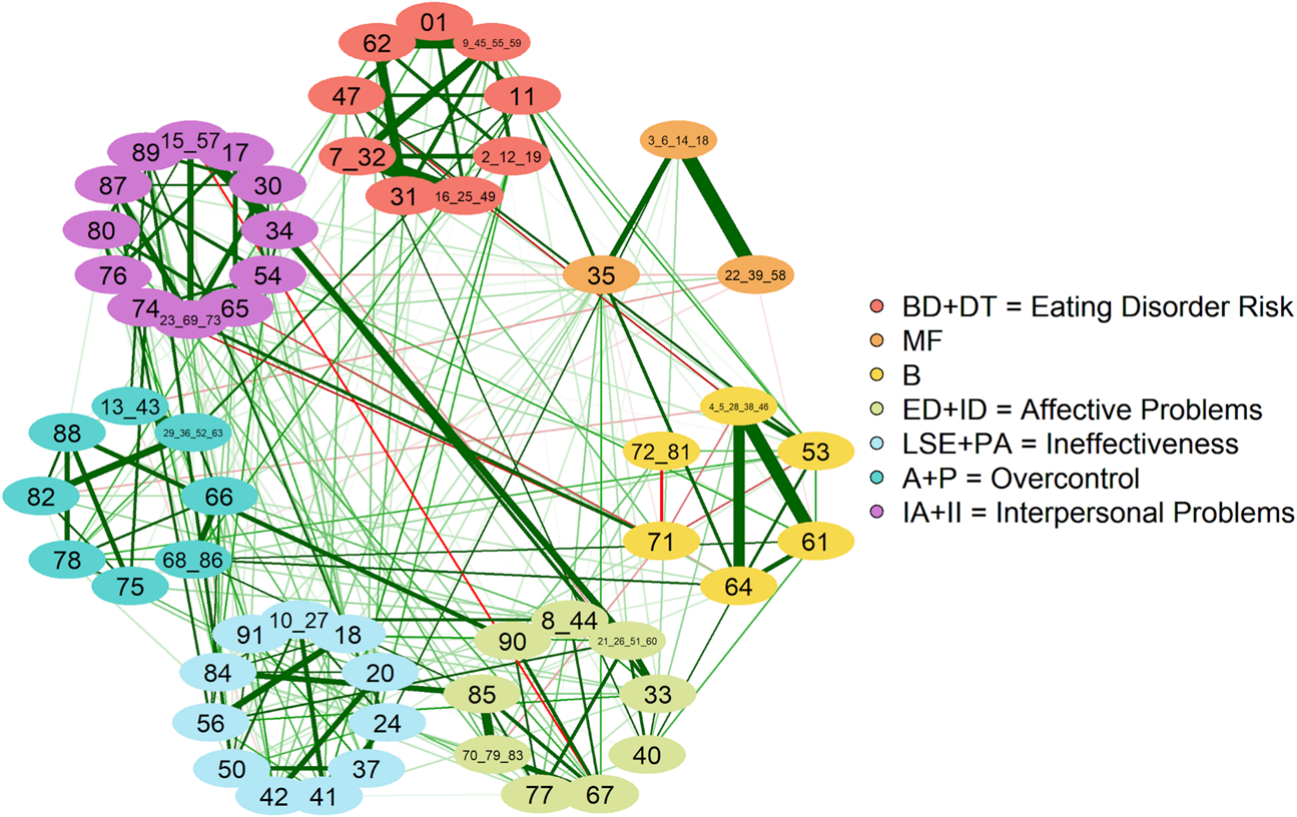
Community structure of the cross-sample network. Nodes have been colored according to the cluster they have been assigned to by the Spinglass community detection algorithm. DT, drive for thinness; B, bulimia; BD, body dissatisfaction; LSE, low self-esteem; PA, personal alienation; II, interpersonal insecurity; IA, interpersonal alienation; ID, interoceptive deficits; ED, emotional dysregulation; P, perfectionism; A, asceticism; MF, maturity fears.

The partition of the median network resulting from the application of the Spinglass algorithm is depicted in Fig. 2.

Seven non-overlapping communities were identified (Table 2). Apart from nodes 72, 81 (ED), and 80 (PA), all the other items were correctly clustered according to the EDI-3 subscale they belong to. Notably, the arrangement of different subscales into communities is consistent with the EDI-3 composite scores, with the sole exception of the Eating Disorder Risk Composite, where the subscale B does not cluster together with BD and DT as would be expected according to the definition given in the manual, and the General Psychological Maladjustment Composite, which however we did not expect to recover since it consists in the combination of all eight psychological scales and thus overlaps with most of the other composites.

**Table 2:** Community composition of the median structure of the cross-sample network according to the Spinglass algorithm.

Community (cross-sample network)	Subscales	Missing items of the subscale	Items from other subscales	Composite score
C1	DT + BD			Eating Disorder Risk
C2	MF			
C3	B		72, 81 (ED)^a^	
C4	ED + ID	72, 81 (ED)		Affective Problems
C5	PA + LSE	80 (PA)		Ineffectiveness
C6	A + P			Overcontrol
C7	II + IA		80 (PA)	Interpersonal Problems

The last column reports the composite score of the EDI-3 questionnaire corresponding to the detected community.

^a^Plus item 71. According to the EDI-3 manual, this item is not classified into any subscale and is not even taken into account during the scoring procedure.

We explored the stability of these dimensions, finding that the most frequent number of detected communities in the cross-sample network is 7 (more precisely, 7 clusters were found in 75.2% of replica samples), which is also the median value of the same distribution with a moderately narrow 95% confidence interval of [6.09,7.90]. However, most dimensions did not result in a stable structural consistency. In fact, only C5 and C3 reported an almost perfect and moderate structural consistency value, respectively (for details see Supplementary Material 1, section 3).

To better understand these results, we analyzed the stability of the problematic items of each dimension. Indeed, we found that the instability was determined by just a few items, since 49 out of 58 of them were instead placed into the same community in at least 70% of the bootstrap replica samples. Among the unstable items, we found again those that were classified into a community inconsistent with the EDI-3 subscale composition, i.e., 72_81 and 80; item 74, which, although being correctly labeled in the median graph, was instead misplaced in the empirical graph (see Table S6); and also the items 71, 89, 84, 40, and 47. Finally, we observed that node 71 was assigned to either of the dimensions B or II + IA in very similar proportions (48% vs 43%), a result that might explain the reason why the EDI-3 manual did not classify it into either of the 12 subscales.

### Diagnosis-specific validation of EDI-3

#### Redundancy analysis

We replicated on the subpopulations of AN and BN patients the same analysis described in section “Redundancy analysis and network estimation” aimed at identifying possible redundant EDI-3 items. In most cases, the results were very similar to those obtained for the cross-sample dataset. Nevertheless, a few discrepancies in the detected redundancy chains are also worth being highlighted (see Table 3). As a result of the redundancy analysis, we finally obtained a subsample of 600 individuals and 55 variables in the case of AN and a subsample of 404 individuals and 61 variables in the case of BN.

**Table 3:** Comparison of redundant EDI-3 items between the AN and BN subpopulations and the cross-sample dataset, as detected via UVA.

Reduced items in the cross-sample dataset	Differences between the reduced items in the AN subpopulation and cross-sample dataset	Differences between the reduced items in the BN subpopulation and cross-sample dataset
4_5_28_38_46		− 28
3_6_14_48		
9_45_55_59	− 55	
21_26_51_60		− 60
29_36_52_63		− 29
2_12_19		− 19
16_25_49		− 25
22_39_58		
7_32	NOT REDUNDANT	+ 25
23_69_73	− 23	
70_79_83	− 70, + 85, + 67	− 70, + 85
8_44		
10_27		NOT REDUNDANT
13_43		
15_57	+ 34	
68_86	+ 66	
72_81		
	+ 31_55_62	+ 74_75^a^
	+ 75_88	+ 31_55

The plus sign (+) in front of item numbers in the second and third columns indicates that such items have been combined with those specified in the first column. Similarly, the minus sign (−) indicates that such items are missing, that is, they are not redundant in the diagnosis-specific datasets.

^a^The redundant pair 74 and 75 detected in the BN subsample is particularly interesting as it is the only one involving items of two different subscales, namely IA and A in this specific scale.

#### Dimension analysis

**Figure 3:**
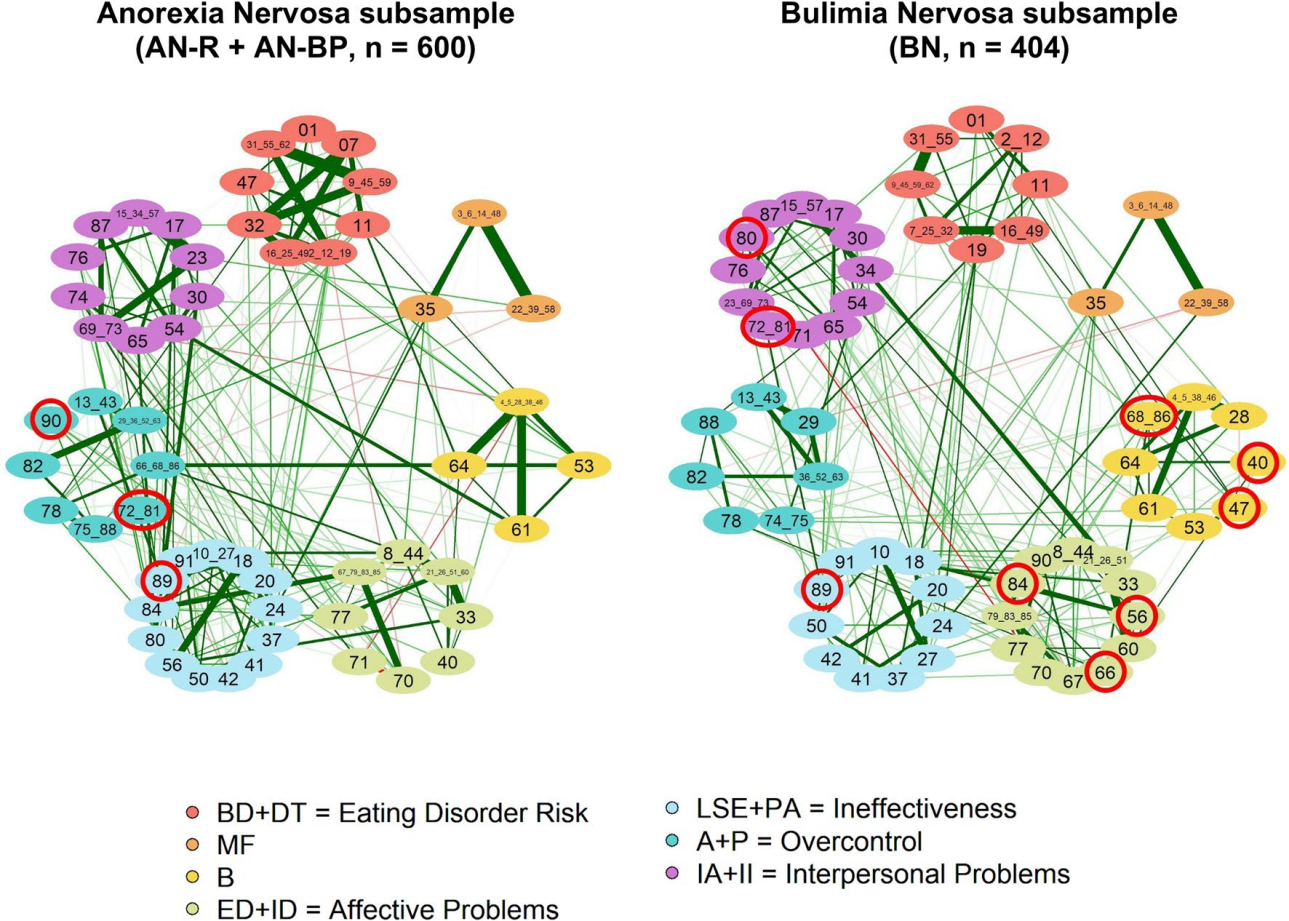
Community structure of the median graphs corresponding to the AN and BN subpopulations. Note that the node composition of the AN and BN networks is slightly different because of a few diverging results of the redundancy analysis. Items assigned to a community different from the corresponding subscales are those hooped with a red circle.

Similarly to the median network structure obtained from the cross-sample data, the AN and BN subpopulations were characterized by a partition into the same seven communities overlapping with the EDI-3 composite scores (Fig. 3). However, as indicated in Table 4, a higher number of items were placed in the “wrong” community, especially in the case of BN.

**Table 4:** Community structure of the median AN and BN networks in comparison to the theoretical dimensional structure of the EDI-3 questionnaire.

Dim	Subscales	AN		BN		Composite Scores
		Missing items of the subscale	Items from other subscales	Missing items of the subscale	Items from other subscales	
C1	DT + BD	–	–	47 (BD)		Eating Disorder Risk
C2	MF	–	–	–	–	–
C3	B	–	–	–	40 (ID), 47 (BD), 68, 86 (A)	–
C4	ED + ID	72, 81, 90 (ED)	^a^	40 (ID), 72, 81 (ED)	56, 84 (PA), 66 (A)	Affective Problems
C5	PA + LSE	–	89 (IA)	56, 80, 84 (PA)	66 (IA)	Ineffectiveness
C6	A + P	–	72, 81, 90 (ED)	66, 68, 86 (A)	–	Overcontrol
C7	II + IA	89 (IA)	–	74 (IA)	80 (PA), 72, 81 (ED)^b^	Interpersonal Problems

^a^Plus item 71.

^b^Plus item 71.

Likewise the cross-sample network, item 71 was mainly connected to other nodes by the negative links, thus resulting in the only item in all networks characterized by a negative one-step and two-step expected influence (EI; i.e., the sum of all edges extending from a given node^64^). In the BN median network, item 72_81 also resulted in a negative (but very close to zero) one-step EI, which however was not reproduced in the second step EI. Interestingly, the assignment of item 71 to a topological community was similarly unstable but sharper in the diagnosis-specific networks compared to the cross-sample. In fact, it was clustered together with ED + ID for 39% of times across the bootstrap replica samples in AN, and together with II + IA for around 48% of times in BN, thus at least doubling the assignment proportion in any other community.

To numerically quantify the agreement of the estimated community structures, we also computed both the normalized and adjusted mutual information scores, where the latter is a variation of the former to account for chance. Table 5 reports the results of the comparison between all pairs of networks under study: the comparison between the two diagnosis-specific networks reveals the greatest disagreement in the community structure (NMI = 0.7664, AMI = 0.7336).

**Table 5:** Normalized and Adjusted Mutual Information score computed between all pairs of networks under study.

Network 1	Network 2	Normalized Mutual Information (NMI)	Adjusted Mutual Information (AMI)
Cross-sample	AN	0.893986	0.879086
Cross-sample	BN	0.795047	0.766241
AN	BN	0.766444	0.733562

Values range from 0 to 1, with 1 corresponding to perfect correlation.

#### Network-based importance evaluation of EDI-3 items

**Figure 4:**
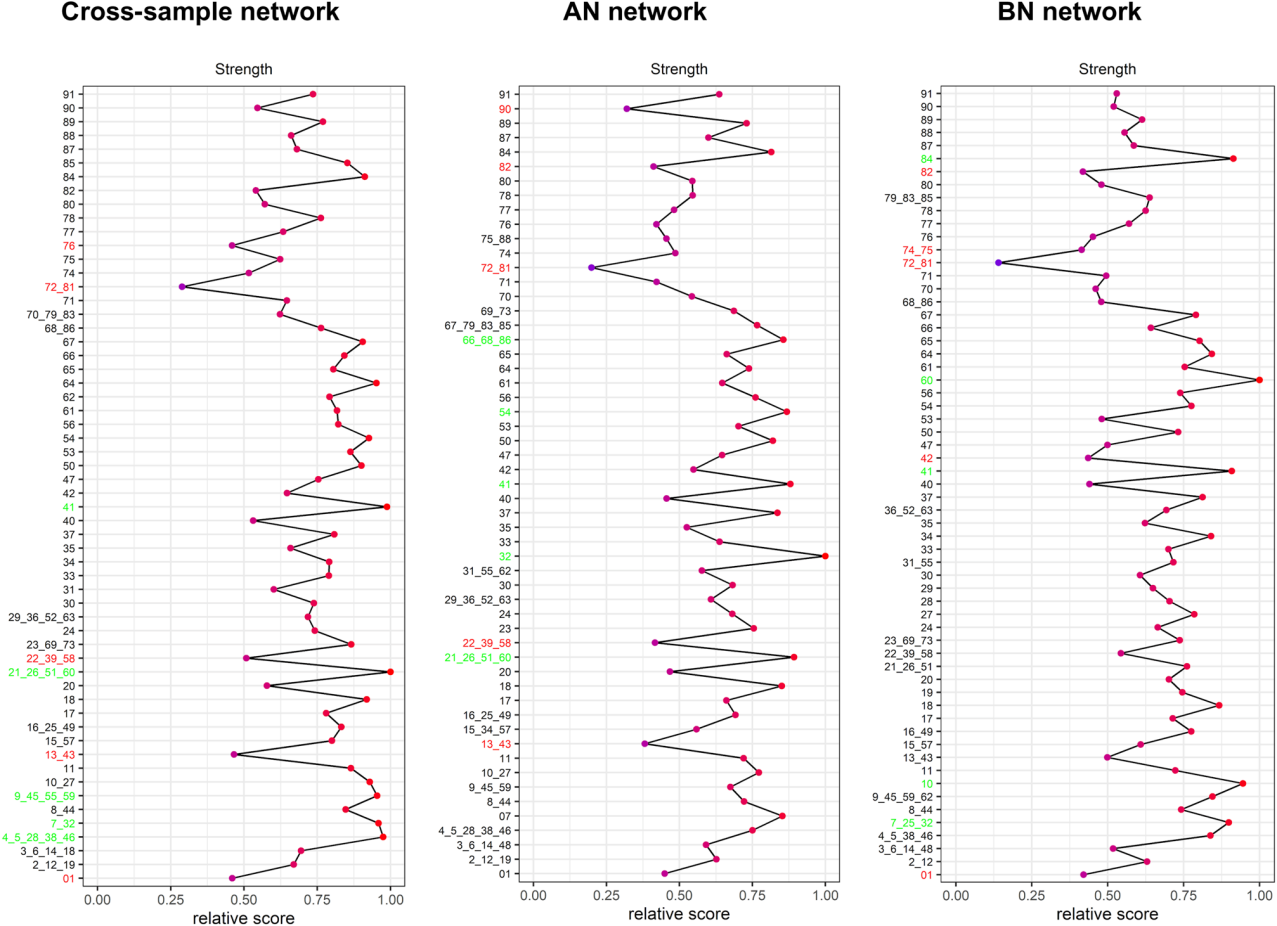
Plots of the strength centrality indices of all items of the (non-redundant) EDI-3 scale, estimated for each of the networks under study (cross-sample, AN, and BN). Nodes in green correspond to the five strongest ones in the corresponding network, whereas the red nodes indicate those with the lowest strength centrality score.

As a final step of our analysis, we identified the nodes with the highest strength centrality, since this index can be easily interpreted as a proxy of the “importance” of certain symptoms or psychological traits in the development and maintenance of EDs^26,31^. Figure 4 shows the results for each of the analyzed networks. There are few nodes particularly central in all the analyzed networks: for example, item 41 *(“I have a low opinion of myself*”) is in the top five of the ranking of the most central nodes in all the cases. On the other hand, some nodes appear to be specific for diagnosis: for instance, item 54 (“*I need to keep people at a certain distance—feel uncomfortable if someone tries to get too close”*) is one of the nodes with highest strength centrality only in AN network, while item 10 (“*I feel ineffective as a person*”) characterizes only the BN network.

## Discussion

Despite the widespread use of the EDI-3, and the large confirmation of its validity, there are some open questions regarding its factor structure^11–13,18^. These questions were often examined following measurement models that derive from a conceptualization of EDs which assumes the property of local independence among symptoms, where the latter are considered manifestations of some common underlying factor and passive receptors of its causal influence (latent variables models). This approach has been largely criticized over the past decades, especially in psychopathology^19–22^. Network models conceptualize EDs as emerging from mutual interactions between symptoms, in which each interaction is based on the pairwise associations between symptoms. The first objective of the present study was to advance the knowledge regarding the validity of the EDI-3 by means of a novel technique based on network analysis.

***RQ1***. The first move towards a scale validation according to the network perspective is redundancy analysis, which also represents the first difference between network and latent variable models. Following the procedure implemented by Christense^55^, UVA detected many redundancy chains across the items of the EDI-3; several new variables were then defined as the combination of items ranging from 2 to 5. This leads to the number of items in the systems decreasing from 91 to 58.

As a second step, dimensionality has been assessed to validate the structure of the scale. The mutual interaction between the resulting 58 items of the EDI-3 yielded seven communities. Two of the communities successfully replicated the primary scales of Bulimia (C3) and Maturity Fears (C2), while four of the communities, although not reflecting the remaining primary scales, retraced the composite scores of Overcontrol (C6, Asceticism and Perfectionism), Ineffectiveness (C5, Personal Alienation and Low Self-Esteem), Affective Problems (C4, Emotional Dysregulation and Interoceptive deficits), and Interpersonal Problems (C7, Interpersonal Insecurity and Interpersonal Alienation). Furthermore, the first community identified (C1) partially reflects the composite score of the Eating Disorder Risk (EDR), including only the primary scales of Drive for Thinness and Body Dissatisfaction, whereas, as stated above, Bulimia forms a community itself. This may be the result of being the only subscale referencing the tendency to engage in an uncontrollable behavior (overeating), whereas DT assesses an extreme desire to be thinner and BD assess discontentment with their body. Further confirmation for this may be in the fact that the Bulimia community also includes items referred to the abuse of alcohol and drugs, which are also considered uncontrollable behavior^65^. This finding is also in line with previous research that identified the Bulimia subscale as a largely specific source of information not contributing much to its overarching composite^66^. To summarize, our results provided support for 2 preliminary subscales: Bulimia and Maturity Fear. Our results also provided support for 4 of the 6 composite scores of the EDI-3 (that in the original conceptualization of the scale are comprised by two or more preliminary subscales), in particular: Overcontrol (Asceticism and Perfectionism), Ineffectiveness (Personal Alienation and Low Self-Esteem), Affective Problems (Emotional Dysregulation and Interoceptive deficits), and Interpersonal Problems (Interpersonal Insecurity and Interpersonal Alienation), as well as a partial support for the composite score of Eating Disorder Risk (Drive for Thinness and Body Dissatisfaction), for which the inclusion of the Bulimia subscale would not be confirmed. Taken together, these results would seem to suggest that the scoring of the Bulimia and the Maturity Fear scales and of the above-mentioned composites can be reliably used in both research and clinical settings. Furthermore, our results would not support the composite score of the Global Psychological Maladjustment, which in the original conceptualization of the scale consists of the summed scores of all 9 of the psychological scales of the EDI-3, however, the use of this composite was already questioned in the scale professional manual^12^. Finally, findings of this study may recommend a more concise version of the scale, where redundant items will be removed, as a reliable instrument that can be easily used with patients and research subjects to measure both eating disorder symptoms and psychological characteristics.

***RQ2.*** As already pointed out, the detection of redundant items of the psychometric assessment tool represents a first novelty introduced by the network perspective. Indeed, not only networks but also latent factor models are negatively impacted by redundancy, as it is a violation of the local independence assumption that often results in poor model fitting and might lead to a misinterpretation of test scores^55^. Hence, the introduction of redundancy analysis as a fundamental step in the psychometric validation workflow is a major contribution to the latent factor model as well.

Secondly, our results of dimension analysis complement the possible outcomes of a traditional validation by offering empirical support for the combination of the specific primary scales in the arrangement of the composite scale proposed by Garner, which in most cases was only supported by weak statistical assumptions^12^.

Most of the dimensions, though, do not show a perfect structural consistency, which refers to the extent to which causally coupled components form a coherent subnetwork within a network^37^. However, when inspecting the loading matrix for each dimension, this seems to depend on only a few items which appear to replicate more in the dimensions identified and less in a second dimension. According to^37^, low structural consistency might not be an issue in presence of multidimensional items, as could be reasonably assumed when interpreting our results. For example, item 89 (“*I know there are people who love me”*) is associated with the Interpersonal Problems dimension in most cases, but its non-negligible correlation with the Ineffectiveness dimension is theoretically well-founded, as the perception of not being loved might also be a sign of personal alienation. In this sense, network analysis complements the results of the latent variable model validation of EDI-3 by adding insights from a multidimensional perspective. Nevertheless, further studies are necessary to develop a cut-off of “high” or “acceptable” structural consistency, as well as more precise pipelines that allow discerning instability related to multidimensionality from that caused by spurious links or indirect influence due to confounding factors^53^. Deep domain knowledge of the investigated psychopathology is likewise fundamental in the interpretation of such results.

***RQ3.*** The validity of the instrument was also investigated separately on specific subpopulations, i.e., patients diagnosed with anorexia nervosa and bulimia nervosa, following the same procedure adopted for the cross-sample patients: identifying redundant nodes, assessing dimensionality, and measuring structural consistency of the dimensions identified through community detection algorithm.

The first step resulted in the identifications of redundant nodes which, in most cases, overlap with those obtained for the cross-sample dataset. Dimensionality assessment confirmed the topological configuration of the seven communities identified in the cross-sample population in both the subsample patients (AN and BN). This result gives general support for the effectiveness of EDI-3 in measuring psychological traits relevant to the development and maintenance of multiple EDs, as such traits show relative stability across diagnostic groups^12,13^. However, some differences emerged at the item level.

Of the twelve items that were differently classified to communities across diagnoses, seven were unstable items in the overall sample as a result of replicating more in the identified dimensions and less in a second cluster. The item *“I feel empty inside (emotionally)”* (56), reflecting a pervasive sense of emotional emptiness, resulted correctly assigned to the dimension of Ineffectiveness (the community that merges the primary scales of low self-esteem and personal alienation) in both the overall sample and the subsample of AN patients, whereas in the BN subpopulations this item seemed to be more densely connected to the Affective Problems dimension. This may suggest that this item evokes the BN patients a sense of emptiness which is more related to the difficulties in the way they interpret and respond to emotional cues. The combined node composed of “*I would like to be in total control of my bodily urges”* and *“I am embarrassed by my bodily urges”* (68_86), which comes from the subscale Asceticism and reflects the tendency to seek virtue through the pursuit of spiritual ideals such as self-discipline, self-denial, self-restraint, self-sacrifice, and control of bodily urge, was assigned to the dimension of Overcontrol in the overall sample and the AN subsample, whereas it was more frequently to the Bulimia dimension in the BN subpopulations. We may hypothesize that the tendency towards uncontrollable overeating typical of BN patients creates a stronger bond with the binge-eating component of the Bulimia dimension rather than with the ascetic behavior. The item *“I feel like I must hurt myself or others”* (90), which comes from the Emotional Dysregulation subscale and reflects the tendency toward mood instability, impulsivity, recklessness, anger, and self-destructiveness, was assigned to the dimension of Affective Problems in both the overall sample and the BN subsample patients, whereas it replicated more often in the Overcontrol dimension in the AN subpopulation. This may suggest that this item evokes in AN patients a sense of pursuit of perfection through self-denial and suffering. A last item, *“I am ashamed of my human weaknesses”* (66), which comes from the Asceticism subscale, was assigned to the dimension of Overcontrol both in the overall sample and the AN patients (in AN subsample it is also redundant with items 68 and 86), whereas in the BN population resulted to be more strongly connected to the dimension of Affective problems. This may suggest that this item evokes in BN patients a sense of shame which for them is more related to the difficulties in managing emotions. However, these differences should be taken with caution because of the remarkable variation within the diagnostic groups on the psychological variables assessed by the EDI-3^13^.

***RQ4.*** Finally, we analyzed the strength centrality index of the EDI-3 symptoms and psychological traits in the overall sample and in the subsample patients. A few symptoms and psychological features were found to be particularly central in all networks analyzed. This is the case of the item *“I have a low opinion of myself”* (41), which refers to low self-esteem, the item *“I have feelings that I find difficult to identify”* (60), which refers to interoceptive deficits and the items *“I am thinking of going on a diet”* (7) and *“I am preoccupied with the desire to be thinner”* (32), which reflects the desire to be thinner, the concern with dieting, preoccupation with weight and an intense fear of weight gain. These items resulted to be the most central not only in the cross-sample network but also in the AN and BN networks. This is important as it generally supports the transdiagnostic theory of EDs, according to which there exist common mechanisms, other than the central cognitive ED specific disturbance, involved in the persistence of different EDs (i.e., clinical perfectionism, core low self-esteem, mood intolerance, and interpersonal difficulties) that also explain the frequent migration of patients across diagnostic groups^41^. Our results replicate many previous findings in support of the transdiagnostic theory^67–71^. Though, only^70,71^ included in their analysis items describing non-specific ED variables and, as a consequence, were able to recognize the central role of the additional transdiagnostic mechanisms as well. On the other hand, the items describing the tendency to abuse alcohol and drugs (72, 81) were found to be the least central nodes in the overall sample, in the AN and BN subsample.

Some differences in the centrality of the items emerged between the diagnosis. Distinctive of AN resulted to be: the item *“I need to keep people at a certain distance (feel uncomfortable if someone tries to get too close)”* (54), which reflects distance, estrangement, and lack of trust in relationships; the items *“I would like to be in total control of my bodily urges”*, *“I am embarrassed by my bodily urges”* and *“I am ashamed of my human weaknesses”* (66, 68, 86), the three reflecting the tendency to seek virtue through the pursuit of spiritual ideals such self-restraint, self-sacrifice and control of bodily urges, and the tendency to view pleasure, relaxing and human weakness as shameful. Central nodes in the BN network were instead: *“I feel ineffective as a person”* (10), which reflected low self-esteem and *“I feel like I am losing out everywhere”* (84), which reflects personal alienation, a pervasive sense of emotional emptiness and aloneness.

As suggested by Borsboom & Cramer^23^, since influential symptoms have strong connections with other symptoms in the network, the probability of such symptoms causing the development of others is high. From this observation, the *centrality hypothesis* is derived, according to which targeting the central symptoms in specific intervention as soon as possible should protect individuals from progressing into disorder^23,24,40^. Despite the wide acceptance of the centrality hypothesis, many remarkable shortcomings have been highlighted concerning many of its theoretical and methodological assumptions that can be hardly completely satisfied in a psychopathological setting, for example, the full interchangeability of symptoms and the inclusion in the model of all variables having a relevant causal effect^52,53^. Nevertheless, we believe that the adoption of a psychometric questionnaire including such a broad spectrum of symptoms and psychological traits, together with the application of a precise redundancy analysis aimed at reducing any source of nuisance variation, allowed us to control the bias produced in our analysis by the limitations underlying the centrality hypothesis^53^. Hence, the results discussed in the current study can indeed offer valid guidance to further empirical studies.

Despite the findings described above, our work has a few limitations as well. First, the lack of a control group precludes the interpretation and statistical comparison between clinical and non-clinical networks. Second, since our data are cross-sectional in nature, the results of this study represent group-level psychopathology networks of EDs at a one-time point only. Longitudinal network analyses would be useful to assess if these results generalize across time and from group to individual. Third, all limitations already described in^13^ due to the demographic composition of the sample and the method employed to impute data apply to our work, too.

As a final note, multidimensional scales, such as EDI-3, often conceal an underlying structure of highly correlated dimensions. In a network setting, this might translate into inconsistencies at the mesoscale level, that is, in the identification of the topological community structure. In fact, most algorithms for community detection, and in particular the one used in this paper, do not allow overlapping nodes between communities. Future studies should address this issue by employing alternative algorithms that let single network variables belong to multiple clusters simultaneously, e.g., the Clique Percolation Method with weights^72–74^.

## Supplementary Information

Supplementary Information 1.

Supplementary Information 2.

## Data Availability

The dataset analyzed during the current study is available in the openICPSR repository at the following URL: https://www.openicpsr.org/openicpsr/project/109443/version/V2/view;jsessionid=21FD8DB3130A73F094AEA1D5AFD21874.
