# Supplementary material for: Network-based validation of the psychometric questionnaire EDI-3 for the assessment of eating disorders
Source: Sci Rep. 2023 Jan 28;13:1578. doi: 10.1038/s41598-023-28743-5 (PMC9884211; doi:10.1038/s41598-023-28743-5)
Supplement: Supplementary file 1 — Supplementary Information 1. [file 41598_2023_28743_MOESM1_ESM.docx]

# Network-based validation of the psychometric questionnaire EDI-3 for the assessment of Eating Disorders

# Supplementary Material 1

# Combined items included in the cross-sample network

| **N.** | **Combined Items (UVA)** | **Subscale** |
| --- | --- | --- |
| 10_27 | 10: I feel ineffective as a person  27: I feel inadequate. | LSE |
| 13_43 | 13: Only outstanding performance is acceptable in my family.  43: My parents expect me to excel. | P |
| 15_57 | 15: I am open about my feelings.  57: I can talk about personal thoughts or feelings. | II |
| 16_25_49 | 16: I am terrified of gaining weight.  25: I exaggerate or magnify the importance of weight.  49: If I gain a pound, I worry that I will keep gaining. | DT |
| 2_12_19 | 2: I think that my stomach is too big.  12: I think my belly is just the right size.  19: I feel satisfied with the shape of my body. | BD |
| 21_26_51_60 | 21: I get confused about what emotion I am feeling.  26: I can clearly identify what emotion I am feeling.  51: When I'm upset, I don't know if I'm sad, scared, or angry.  60: I have feelings that I find difficult to identify. | ID |
| 22_39_58 | 22: I would rather be an adult than a child.  39: I feel happy that I am not a child anymore.  58: The best years of your life are when you become an adult. | MF |
| 23_69_73 | 23: I can communicate with others easily.  69: I feel relaxed in most group situations.  73: I am dynamic with most people. | II |
| 29_36_52_63 | 29: As a child, I tried very hard to avoid disappointing my parents and teachers.  36: I hate being less than best at things.  52: I feel that I must do things perfectly or not do them at all.  63: I have extremely high goals. | P |
| 3_6_14_48 | 3: I wish that I could return to the security of childhood.  6: I wish that I could be younger.  14: The happiest time in life is when you are a child.  48: I feel that people are happiest when they are children. | MF |
| 4_5_28_38_46 | 4: I eat when I am upset.  5: I binge eat  28: I have gone on eating binges where I felt that I could not stop.  38: I think about bingeing (overeating).  46: I eat moderately in front of others and stuff myself when they are gone. | B |
| 68_86 | 68: I would like to be in total control of my bodily urges.  86: I am embarrassed by my bodily urges. | A |
| 7_32 | 7: I'm thinking of going on a diet.  32: I am preoccupied with the desire to be thinner. | DT |
| 70_79_83 | 70: I say things impulsively that I regret having said.  79: I am prone to outbursts of anger or range.  83: Others would say that I get irritated easily. | ED |
| 72_81 | 72: I have to be careful with my tendency to abuse drugs  81: I have to be careful with my tendency to abuse alcohol. | ED |
| 8_44 | 8: I get frightened when my feelings are too strong.  44: I worry that my feelings will get out of control. | ID |
| 9_45_55_59_62 | 9: I think that my thighs are too large.  45: I think my hips are too large.  55: I think that my thighs are just the right size.  59: I think my buttocks are too big.  62: I think my hips are the right width. | BD |

Table S 1. The 17 items listed in the table above correspond to the items obtained by combination of redundant items as detected by the UVA algorithm on the cross-sample network (see Section 3.1 of the main text).For each item, its identification number, its description, and the subscale it belongs to are given.

# Redundancy analysis: Goldbricker vs UVA

The 91 symptoms and psychological traits assessed by the EDI-3 items were inspected to reveal any possible redundant node. Apart from the UVA technique proposed in the main text, we present here a comparison with Goldbricker [[1]](https://paperpile.com/c/cAJceU/ZYwb), a method that determines whether a pair of correlations to a third variable are significantly different from each other by first calculating the proportion of correlations which are significantly different for each different pair of nodes and then by defining all those pairs falling below an arbitrary threshold as redundant.

The *goldbricker()* function was run with 0.25 as the threshold proportion and 0.1 as the *p*-value threshold parameter. A list of 39 “bad pairs” was returned. However, many nodes were included in multiple pairs, therefore only 12 of them could actually be combined (see Table S 2 for the full list). We decided to reduce the redundant nodes by removing one item from each pair instead of using PCA to keep the same 0-4 ordinal scale throughout the whole dataset. Hence, the final network was composed of a total of 79 symptoms (i.e., nodes). In comparison to UVA, Goldbricker detected far fewer pairs of redundant nodes. In particular, 9 out of 12 pairs were identified as redundant by both algorithms, while 3 out of 12 only by Goldbricker ( $12 \leftrightarrow55$, $16 \leftrightarrow7$ and $2 \leftrightarrow9$).

| **Bad Pairs** | | **Threshold** | **Selected Item** |
| --- | --- | --- | --- |
| 57: I can talk about personal thoughts or feelings (II) | 15: I am open about my feelings (II) | 0.04494382 | 57 |
| 51: When I'm upset, I don't know if I'm sad, scared, or angry. angry (ID) | 21: I get confused about what emotion I am feeling (ID) | 0.05617978 | 51 |
| 55: I think that my thighs are just the right size. (BD) | 12: I think my belly is just the right size (BD) | 0.08988764 | 55 |
| 48: I feel that people are happiest when they are children (MF) | 14: The happiest time in life is when you are a child (MF) | 0.10112360 | 14 |
| 28: I have gone on eating binges where I felt that I could not stop (B) | 4: I eat when I am upset (B) | 0.11235955 | 4 |
| 27: I feel inadequate (LSE) | 10: I feel ineffective as a person (LSE) | 0.12359551 | 10 |
| 16: I am terrified of gaining weight (DT) | 7: I'm thinking of going on a diet (DT) | 0.13483146 | 16 |
| 9: I think that my thighs are too large (BD) | 2: I think that my stomach is too big (BD) | 0.14606742 | 2 |
| 46: I eat moderately in front of others and stuff myself when they are gone (B) | 5: I binge eat (B) | 0.14606742 | 46 |
| 44: I worry that my feelings will get out of control (ID) | 8: I get frightened when my feelings are too strong (ID) | 0.15730337 | 44 |
| 58: The best years of your life are when you become an adult (MF) | 39: I feel happy that I am not a child anymore (MF) | 0.15730337 | 39 |
| 52: I feel that I must do things perfectly or not do them at all (P) | 36: I hate being less than best at things (P) | 0.20224719 | 52 |

Table S 2. List of redundant nodes identified via the goldbricker function. For each pair, the proportion of significantly different correlation is given, together with the node that is kept in the final network.

# Internal structural consistency of EDI-3

The internal consistency of psychometric questionnaires is predominantly quantified by means of Cronbach’s alpha $\alpha_{C}$ [[2]](https://paperpile.com/c/cAJceU/kFkr). Since this measure requires unidimensional data, we computed it separately on each of the twelve EDI-3 subscales, as well as for each of the six composite scores (see Table S 3).

| **Subscale** | $\alpha_{C}$ | 2.5% | 97.5% |
| --- | --- | --- | --- |
| DT | 0.593 | 0.557 | 0.625 |
| BD | 0.740 | 0.718 | 0.759 |
| B | 0.695 | 0.669 | 0.720 |
| MF | 0.674 | 0.646 | 0.700 |
| II | 0.589 | 0.552 | 0.622 |
| IA | 0.567 | 0.530 | 0.603 |
| ED | 0.608 | 0.574 | 0.638 |
| ID | 0.689 | 0.664 | 0.712 |
| LSE | 0.715 | 0.686 | 0.741 |
| P | 0.654 | 0.623 | 0.682 |
| PA | 0.640 | 0.606 | 0.671 |
| A | 0.720 | 0.695 | 0.742 |
| **Composite score** | $\alpha_{C}$ | 2.5% | 97.5% |
| EDRC | 0.868 | 0.858 | 0.878 |
| IC | 0.797 | 0.779 | 0.815 |
| IPC | 0.723 | 0.702 | 0.742 |
| APC | 0.786 | 0.770 | 0.802 |
| OC | 0.802 | 0.786 | 0.818 |
| GPMC | 0.941 | 0.936 | 0.945 |

Table S 3. Values of Cronbach’s alpha computed on the full sample of 1206 individuals and 91 variables for each EDI-3 subscale and each composite score.

From a network perspective, the internal consistency of a psychometric network (e.g., computed via Gaussian Graphical Model with LASSO regularization), needs to be assessed differently. Christensen et al. [[3]](https://paperpile.com/c/cAJceU/UUi8E/?noauthor=1) suggested to estimate the so-called *structural consistency*, which is defined as the extent to which items in a dimension are homogeneous and interrelated given the multidimensional structure of the questionnaire. Hence, following the approach in [[3]](https://paperpile.com/c/cAJceU/UUi8E/?noauthor=1), we first run a 500 iteration resampling bootstrap procedure ([[4], [5]](https://paperpile.com/c/cAJceU/TYYy+medK)), next we computed the structural consistency corresponding to the Spinglass clustering of the empirical graph and finally the item stability of the same (Table S 6, last two columns). Importantly, note that, for the rest of this section, the focus is on the *empirical graph*, i.e., the GLASSO network estimated from the original dataset, whereas the validation analysis carried out in the main text was based on the *median graph*, that is, a graph with same nodes as the empirical one, but with edge weights corresponding to the median values of the edge weights estimated across all bootstrap replica samples.

The summary statistics corresponding to the bootstrap replica samples of the cross, AN and BN network are displayed in Table S 4 and Table S 5.

| Network | # Boots | Median | Standard deviation | Lower 95% CI | Upper 95% CI | Lower (2.5%) quantile | Upper (97.5%) quantile |
| --- | --- | --- | --- | --- | --- | --- | --- |
| Cross | 500 | 7 | 0.460421 | 6.095396 | 7.904604 | 7 | 8 |
| AN | 500 | 7 | 0.638811 | 5.744908 | 8.255092 | 6 | 8 |
| BN | 500 | 8 | 0.739864 | 6.546367 | 9.453633 | 6 | 9 |

Table S 4. Descriptive statistics of the EDI-3 dimensions detected across all bootstrap replicate samples, divided by network.

| # Dimensions | Cross | AN | BN |
| --- | --- | --- | --- |
| 6 | 0.022 | 0.134 | 0.028 |
| 7 | 0.752 | 0.596 | 0.358 |
| 8 | 0.224 | 0.262 | 0.486 |
| 9 | 0.002 | 0.008 | 0.130 |
| 10 |  |  | 0.006 |

Table S 5. Frequency of EDI-3 number of dimensions detected across all bootstrap replicate samples.

In the case of the cross-sample and AN empirical networks, the number of detected dimensions reflects that of the corresponding median network. Moreover, with regards to both AN and BN, the partition into the number of dimensions suggested by the median value might be unstable, as demonstrated by the high standard deviation.

In general, to understand the source of instability, one can first look at the structural consistency of each dimension to find those provoking the instability, and then dig further into each unstable dimension to highlight which of its items are indeed characterized by low stability. In the following subsections, we show how this analysis has been carried out in the case of the cross-sample network and BN and AN networks, respectively.

## Item stability of the cross-sample network

In operational terms, the structural consistency of a given graph partition is defined as the proportion of times each empirically derived dimension is recovered from the replicate bootstrap samples with an identical item composition. Instead, the item stability index informs about the proportion of times each item is detected in each empirically derived dimension across the replicate samples. Importantly, nodes with low stability scores might not be indicators of structural inconsistency, but they could rather be interpreted as multidimensional items. We referred to the loading matrix to adequately clarify the item stability scores.

| **Community (empirical graph)** | Subscales | Missing items of the subscale | Items from other subscales | Structural consistency | Avg. item stability |
| --- | --- | --- | --- | --- | --- |
| C1 | DT + BD |  |  | 0.416 | 0.973 |
| C2 | MF |  |  | 0.426 | 0.856 |
| C3 | B |  | 72, 81 (ED)* | 0.674 | 0.895 |
| C4 | ED + ID | 72, 81 (ED) |  | 0.224 | 0.917 |
| C5 | PA + LSE | 80 (PA) |  | 0.926 | 0.886 |
| C6 | A + P |  | 74 (IA) | 0.564 | 0.808 |
| C7 | II + IA | 74 (IA) | 80 (PA) | 0.422 | 0.890 |

Table S 6. Community composition of the empirical network according to the Spinglass algorithm. The structural consistency column reports the proportion of times each community in the empirical graph was exactly recovered from the replicate bootstrap samples, while the average item stability column indicates the average number of times each variable is estimated in the same dimension as originally estimated in the empirical graph.

* Plus item 71

As a first observation, one can note that the only difference in the community structure of the empirical compared to the median graph is the placement of item 74 (*I feel trapped in relationships*), which was improperly assigned to A+P instead of II+IA. Nevertheless, continuing our analysis, we found this positioning to be very unstable, meaning that it showed in only 24% of cases across the replica samples, in contrast to the 67% of correct assignments. Refer to Supplementary material 2 (sheet cross_item_stability) for the full table with the replication proportions of each item in all dimensions.

Continuing the examination of the empirical cross-sample network, we found that, apart from node 74, many other edges were causing moderate structural inconsistencies, as illustrated in Figure S 1. We thus analyzed their replication proportions across dimensions to find out the reason for their low stability, which we mainly interpreted as a manifestation of their cross-dimensional nature. In the following, we list the multiple memberships of those having item stability below the acceptable threshold of 0.7 [[5]](https://paperpile.com/c/cAJceU/medK):

- 72_81 (*I have to be careful with my tendency to abuse drugs or alcohol*; ED) → 45% in B, 29% in IA + II, 17% in ED + ID.
- 71 (*I go out of my way to experience pleasure*; - ) → 48% in B, 43% in II + IA.
- 89 (*I know there are people who love me*; IA) → 57% in II + IA, 41% in LSE. + PA.
- 84 (*I feel like I am losing out everywhere*; PA) → 58% in LSE + PA; 33% in ED + ID.
- 40 (*I get confused as to whether or not I am hungry*, ID) → 59% in ED + ID; 31% in B.
- 80 (*I feel that people give me the credit I deserve*; PA) → 69% in II + IA, 28% in LSE + PA.
- 47 (*I feel bloated after eating a normal meal*; BD) → 69% in BD + DT; 29% in B.


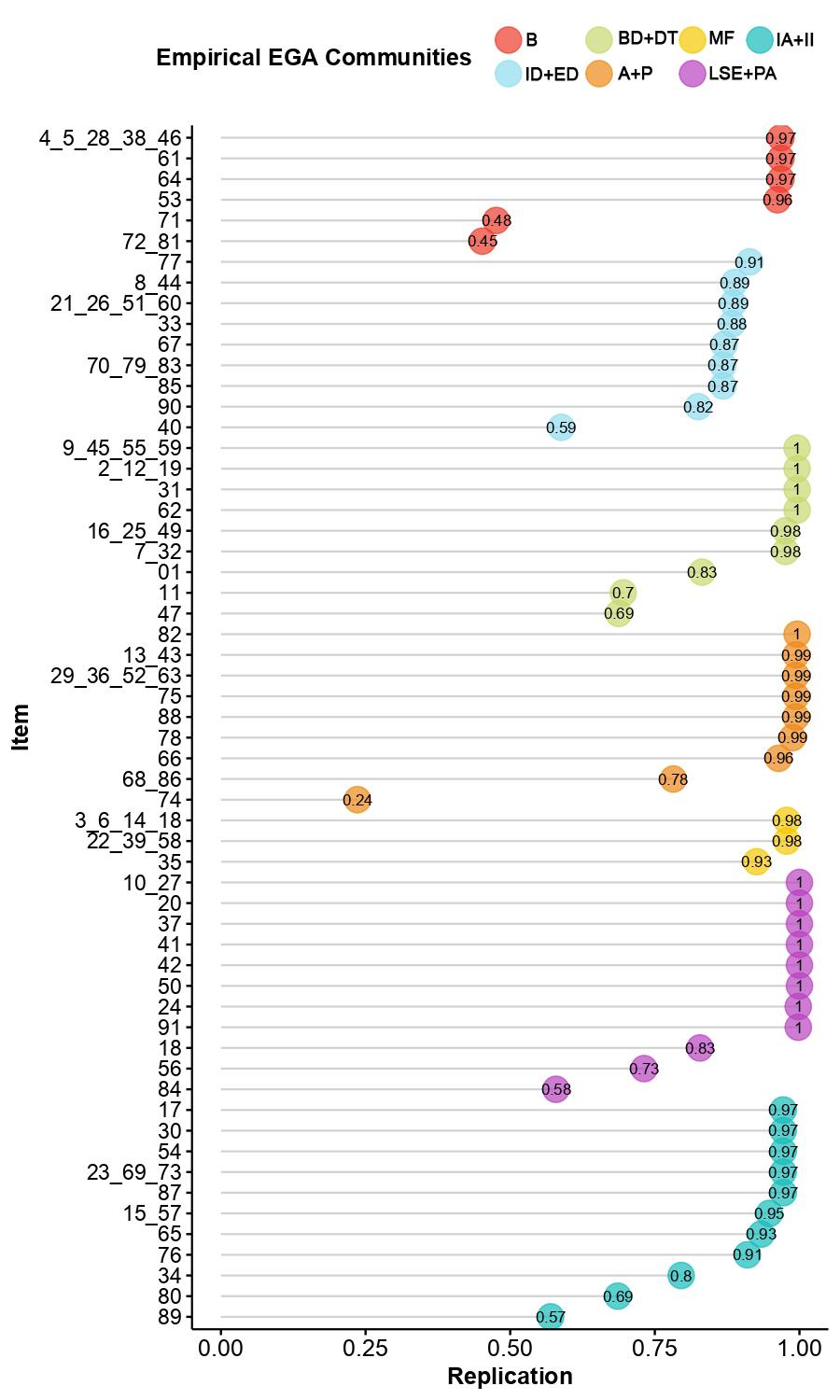


Figure S 1. Item stability of the cross-sample network: number of times each item replicated within the same dimension as the one assigned in the empirical graph. For example, the item 74 was assigned to the community A+P in the empirical graph; however, it replicated within this community only 24% of times across all replicate samples and 67% of times in the correct community IA+II, which is also the one it was assigned to in the median graph described in the main text (the numbers are taken from Supplementary material 2).

## Item stability of the diagnosis-specific networks

Replicating the procedure employed for the cross-sample network, we estimated the structural consistency and the item stability of the two diagnosis-specific networks, namely AN (Anorexia Nervosa, Table S 7) and BN (Bulimia Nervosa, Table S 8).

| **Community (AN)** | Subscales | Missing items of the subscale | Items from other subscales | Structural consistency | Avg. item stability |
| --- | --- | --- | --- | --- | --- |
| 1 | DT + BD |  |  | 0.962 | 0.792 |
| 2 | MF |  |  | 0.842 | 0.909 |
| 3 | B |  |  | 0.716 | 0.969 |
| 4 | ED + ID |  | * | 0.682 | 0.899 |
| 5 | PA + LSE |  | 89 (IA) | 0.226 | 0.804 |
| 6 | A + P |  |  | 0.380 | 0.818 |
| 7 | II + IA | 89 (IA) |  | 0.078 | 0.831 |

Table S 7. Community composition of the empirical AN network according to the Spinglass algorithm. The structural consistency column reports the proportion of times each community in the empirical graph was exactly recovered from the replicate bootstrap samples, while the average item stability column indicates the average number of times each variable is estimated in the same dimension as originally estimated in the empirical graph. * Plus item 71

| **Community (BN)** | Subscales | Missing items of the subscale | Items from other subscales | Structural consistency | Avg. item stability |
| --- | --- | --- | --- | --- | --- |
| 1 | DT + BD | 47 (BD) |  | 0.110 | 0.814 |
| 2 | MF |  |  | 0.014 | 0.638 |
| 3 | B |  | 40 (ID) +  47 (BD) +  68, 86 (A) | 0.736 | 0.755 |
| 4 | ID + ED | 40 (ID) | 91 (PA) | 0.376 | 0.686 |
| 5 | PA + LSE | 80, 91 (PA) | 66 (A) * | 0.570 | 0.893 |
| 6 | A + P | 66, 68, 86 (A) | 74 (IA) | 0.264 | 0.777 |
| 7 | II + IA | 74 (IA) | 80 (PA) | 0.128 | 0.842 |

Table S 8. Community composition of the empirical BN network according to the Spinglass algorithm. The structural consistency column reports the proportion of times each community in the empirical graph was exactly recovered from the replicate bootstrap samples while the average item stability column indicates the average number of times each variable is estimated in the same dimension as originally estimated in the empirical graph.

* Plus item 71

**AN Network.** As suggested by Figure S 2, the dimensions LSE+PA, ED+ID and II+IA in the AN network show from moderate to high instabilities. In the following, we list the replication proportions across multiple dimensions of the less stable items:

- 72_81 (*I have to be careful with my tendency to abuse drugs or alcohol*; ED) → 34% in ID+ED, 16% in 8, 16% in A+P, 14% in B.
- 90 (*I feel that I really know who I am*; PA) → 36% in A+P, 33% in ID+ED, 14% in C8.
- 71 (*I go out of my way to experience pleasure*; -) → 39% in ID+ED, 18% in 8, 17% in II+IA, 16% in B.
- 74 (*I feel trapped in relationships*; IA) → 47% in II+IA, 20% in C8, 13% in ID+ED.
- 89 (*I know there are people who love me*; IA) → 47% in LSE+PA, 47% in II+IA.
- 80 (*I feel that people give me the credit I deserve*; PA) → 55% in LSE+PA, 33% in II+IA.
- 70 (*I say things impulsively that I regret having said*; ED) → 58% in ID+ED, 19% in C8.
- 56 (*I feel empty inside emotionally*; PA) → 58% in LSE+PA, 30% in ID+ED.
- 65 (*People I really like end up disappointing me*; IA) → 65% in II+IA, 18% in C8.
- 84 (*I feel like I am losing out everywhere*; PA) → 66% in LSE+PA, 21% in ID+ED.


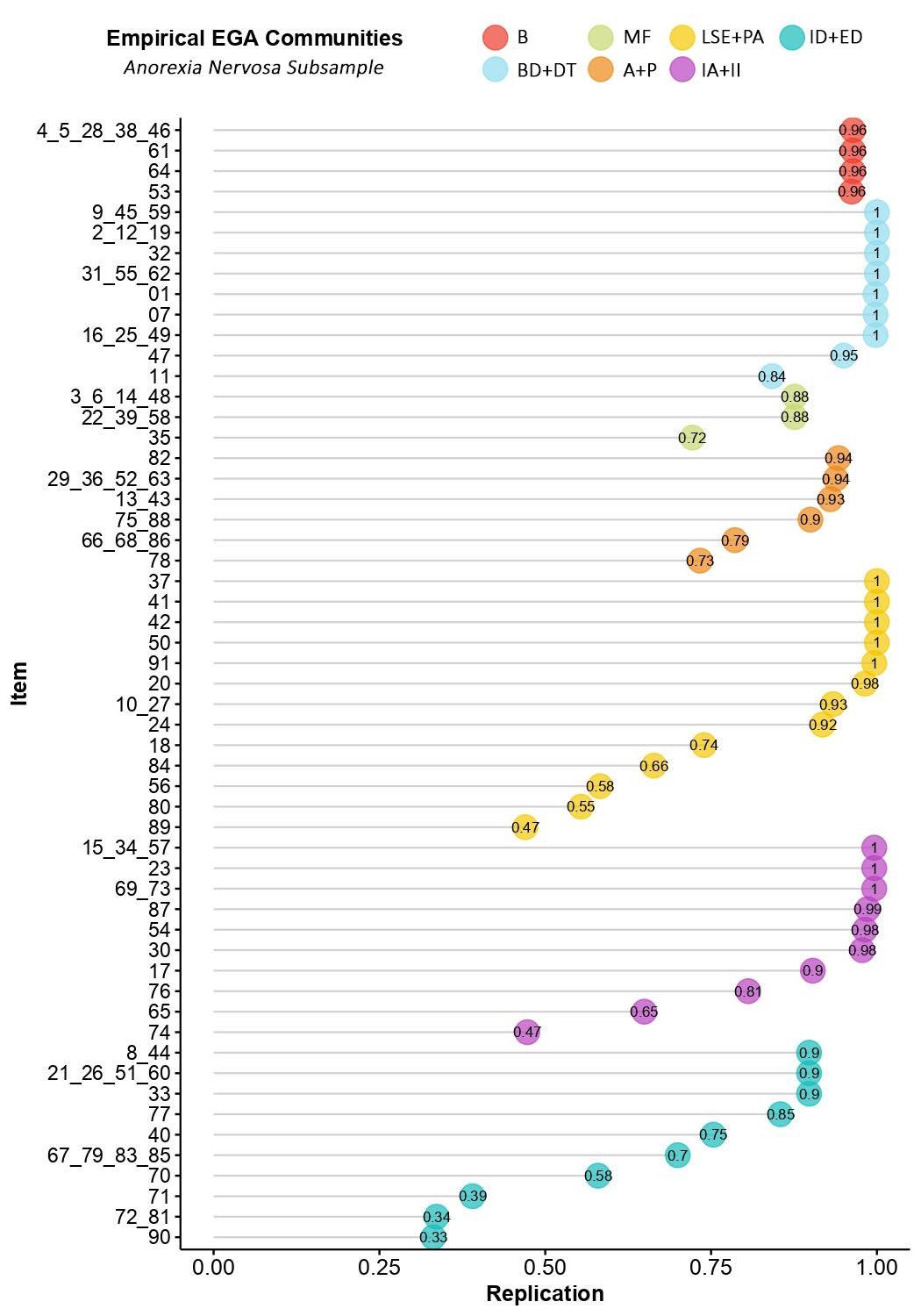


Figure S 2. Item stability of the AN subsample network: number of times each item replicated within the same dimension as the one assigned in the empirical graph. For the complete table of proportion of times each item replicated in each of the empirical dimensions refer to **Supplementary Material 2** (sheet an_item_stability).

**BN Network.** Finally, we analyzed the stability of dimensions and items of the BN network (Figure S 3). Compared to the previous cases, many more instabilities were detected, with 21 out of 61 items having an item stability score below the minimum threshold of 0.7. In particular, only the dimension DT+BD did not show any instability, whereas MF and II+IA had one (35) and two (80, 89) items, respectively, just below the threshold. In the dimension LSE+PA, the most unstable items were:

- 56 (I feel empty inside (emotionally); PA) → 40% in LSE+PA, 22% in C8, 20% in ID+ED, 11% in II+IA.
- 84 (*I feel like I am losing out everywhere*; PA) → 31% in C8, 26% in LSE+PA, 21% in ID+ED.
- 66 (*I am ashamed of my human weaknesses;* A) → 26% in C8, 19% in LSE+PA, 17% in B, 16% in A+P, 15% in ID+ED.

In the dimension ID+ED, only one third of the items were stable. Those performing worstly were:

- 72_81 (*I have to be careful with my tendency to abuse drugs or alcohol*; ED) → 37% in ID+ED, 27% in II+IA, 20% in C8.
- 71 (*I go out of my way to experience pleasure*; -) → 48% in II+IA, 24% in ED+ID, 19% in C8
- 91 (*I feel that I really know who I am*; PA) → 60% in LSE+PA, 26% in ED+ID.

In the dimension A+P a couple of items were particularly unstable:

- 78 (*Eating for pleasure is a sign of moral weakness*; A)→ 46% in A+P, 16% in C8, 12% in BD+DT, 10% in ED+ID.
- 74_75 (*I feel trapped in relationships*; IA - *Self-denial makes me feel stronger spiritually*; A) → 41% in A+P, 23% in ED+ID, 19% in C8.

Finally, in the dimension B, only one item has a very low stability:

- 47 (*I feel bloated after eating a normal meal*; BD) → 51% in BD+DT, 28% in B, 13% in A+P.


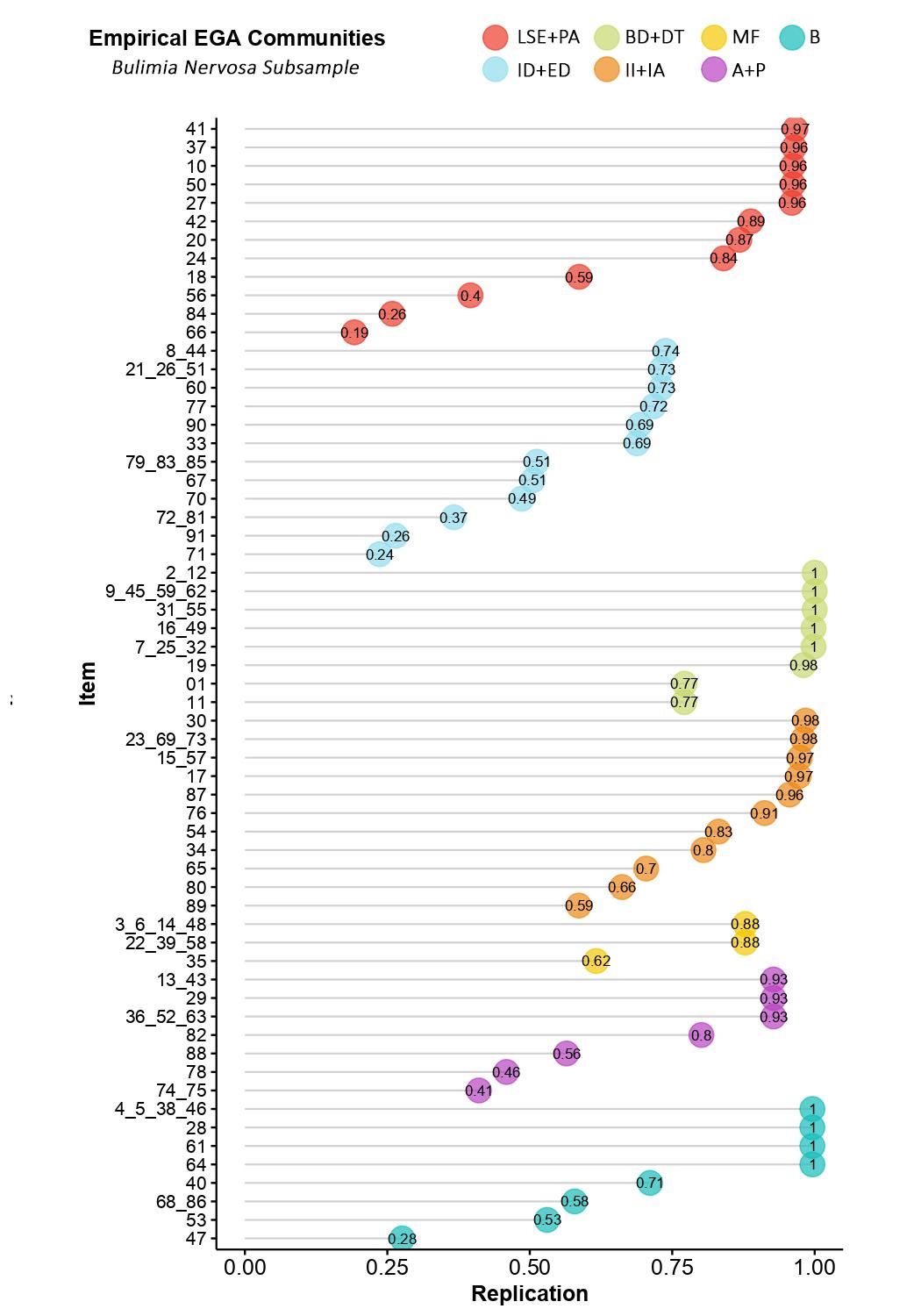


Figure S 3. Item stability of the BN subsample network: number of times each item replicated within the same dimension as the one assigned in the empirical graph. For the complete table of proportion of times each item replicated in each of the empirical dimensions refer to Supplementary Material 2 (sheet bn_item_stability).

# Stability of centrality indices

The stability of strength and expected influence centrality indices has been assessed by means of the case-dropping bootstrap proposed in [[6]](https://paperpile.com/c/cAJceU/H1HV). As depicted in Figure S 4 the diagnosis-specific subpopulations resulted in a lower stability compared to the cross-sample due to their reduced sample size. Although both strength and expected influence had a CS-coefficient above the suggested threshold for reliable interpretations in the cross and AN sample (see Table S 9), only strength was sufficiently stable in BN. Hence, in the main text we limit our discussion only to this centrality measure.

| Case-dropping bootstrap to assess the stability of centrality indices | | |
| --- | --- | --- |
| Cross-sample | AN subsample | BN subsample |
| 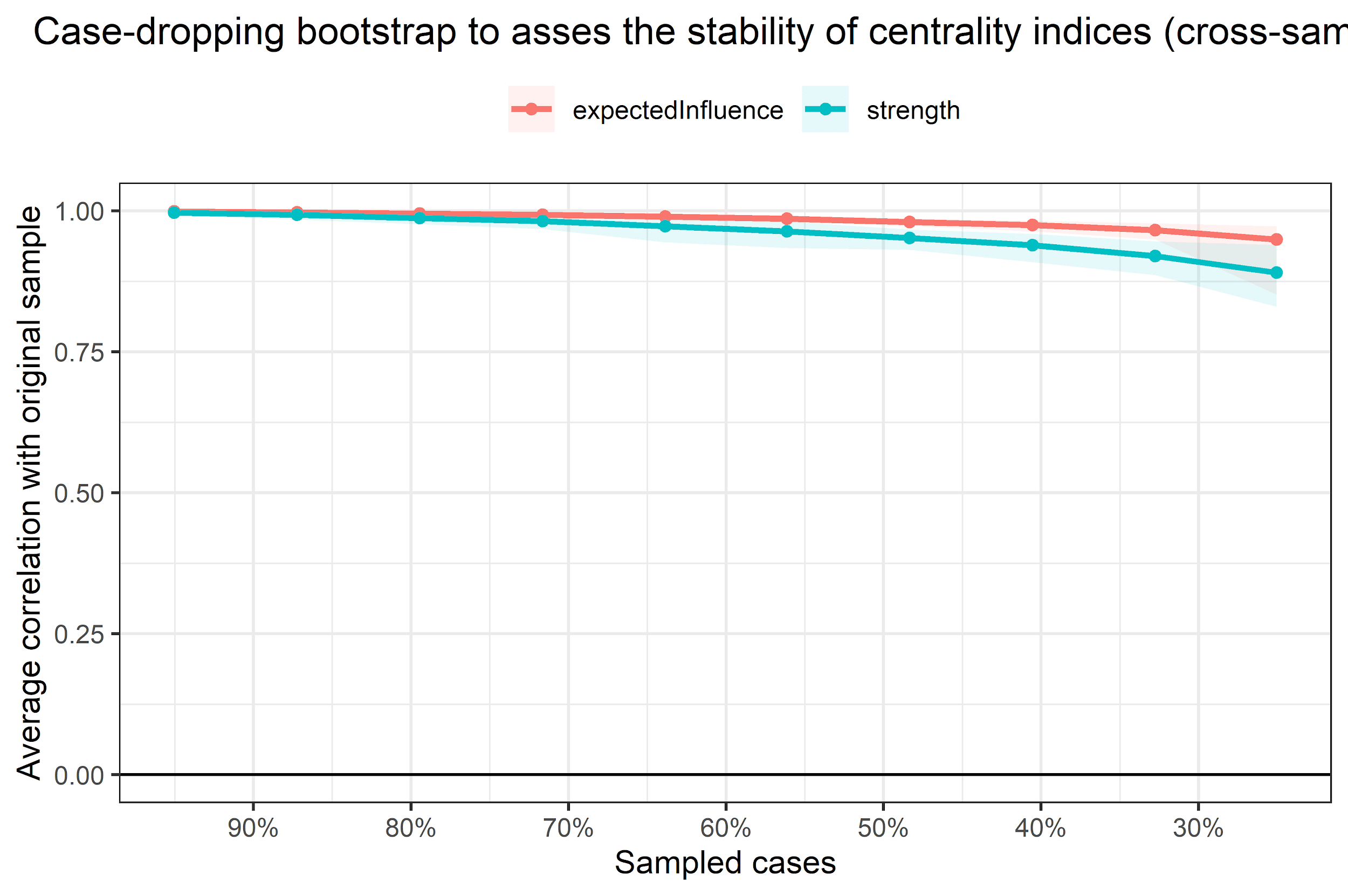 | 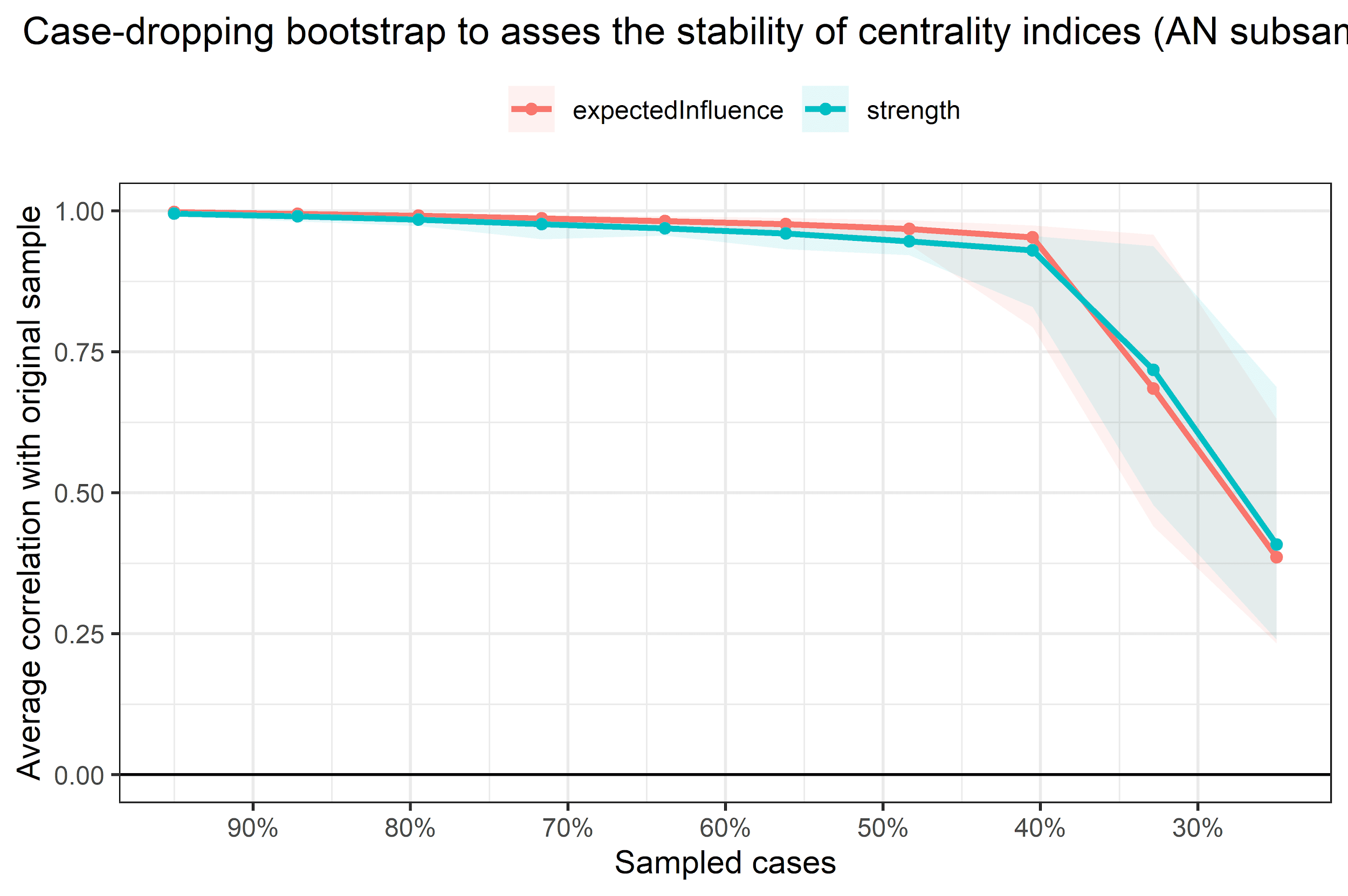 | 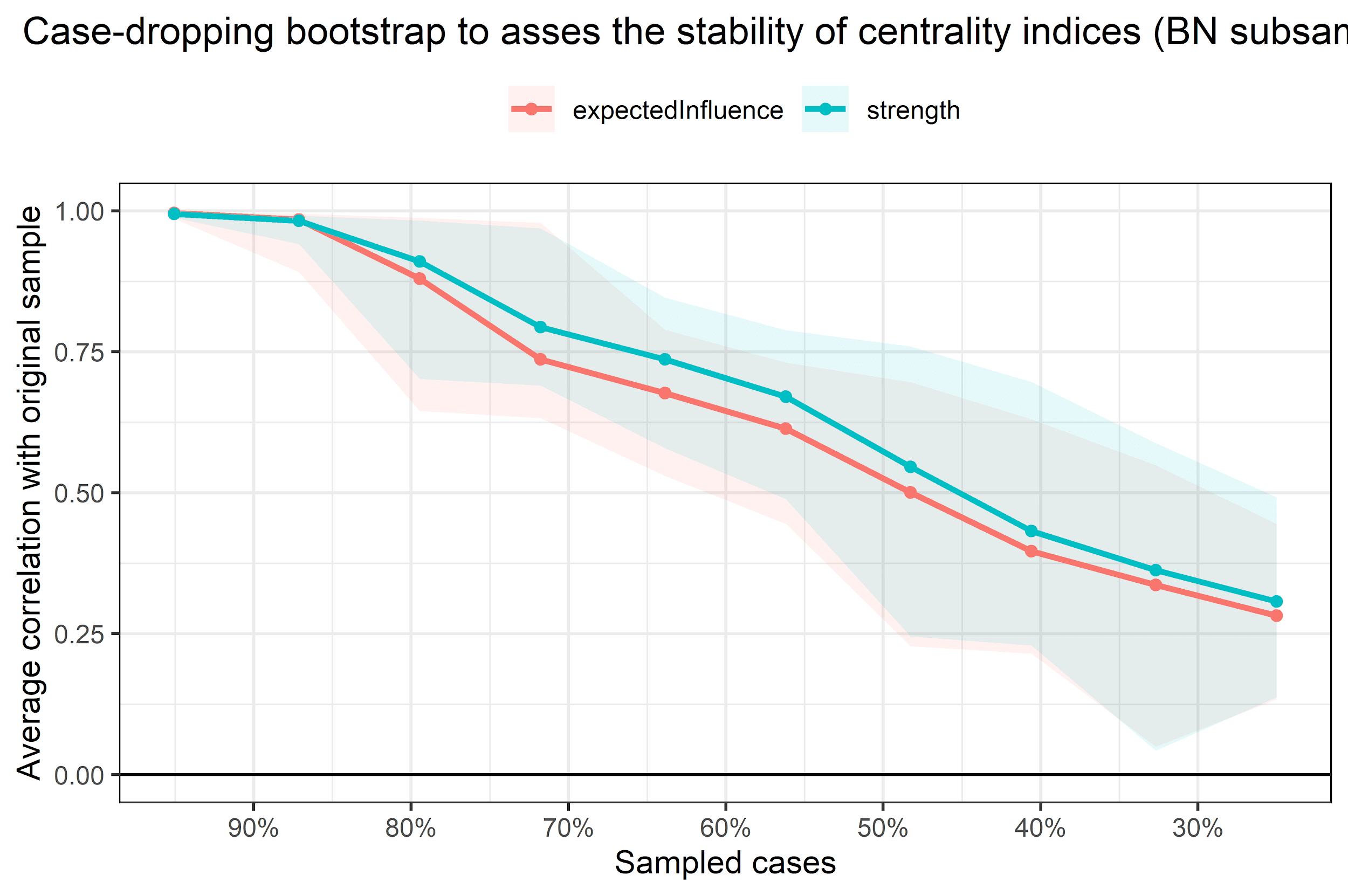 |

Figure S 4. Case-dropping bootstrap

|  | **CROSS** | **AN** | **BN** |
| --- | --- | --- | --- |
| **Strength** | 0.75 | 0.595 | 0.282 |
| **Expected influence** | 0.75 | 0.595 | 0.129* |

Table S 9. Correlation Stability Coefficient (CS) of the three datasets with respect to strength and expected influence centrality indices.

* Below the suggested minimum threshold of 0.25 [6]

# Reference List

[1] [P. J. Jones, “Networktools: Tools for identifying important nodes in networks,” *R package version*, vol. 1, no. 0, pp. 10–1155, 2018.](http://paperpile.com/b/cAJceU/ZYwb)

[2] [L. J. Cronbach, “Coefficient alpha and the internal structure of tests,” *Psychometrika*, vol. 16, no. 3. pp. 297–334, 1951. doi:](http://paperpile.com/b/cAJceU/kFkr) [10.1007/bf02310555.](http://dx.doi.org/10.1007/bf02310555.)

[3] [A. P. Christensen and H. Golino, “A psychometric network perspective on the validity and validation of personality trait questionnaires,” *European Journal of*, 2020, [Online]. Available:](http://paperpile.com/b/cAJceU/UUi8E) <https://journals.sagepub.com/doi/abs/10.1002/per.2265?casa_token=NKrBXPRLAKwAAAAA:cD_JJFC7ZOF39BhlZ7a6e-6SLj5a2u4F6RtTz6df04yTinzPurbtGh5s8tSnEkLLoHWl1WC2IG8lB0l0>

[4] [Golino, Christensen, and Moulder, “EGAnet: Exploratory graph analysis: A framework for estimating the number of dimensions in multivariate data using network psychometrics,” 2021.](http://paperpile.com/b/cAJceU/TYYy)

[5] [A. P. Christensen and H. Golino, “Estimating the stability of psychological dimensions via bootstrap exploratory graphs analysis: A monte carlo simulation and tutorial,” *Psych*, vol. 3, no. 3, pp. 479–500, 2021, doi:](http://paperpile.com/b/cAJceU/medK) [10.3390/psych3030032.](http://dx.doi.org/10.3390/psych3030032.)

[6] [S. Epskamp, D. Borsboom, and E. I. Fried, “Estimating psychological networks and their accuracy: A tutorial paper,” *Behav. Res. Methods*, vol. 50, no. 1, pp. 195–212, 2018, doi:](http://paperpile.com/b/cAJceU/H1HV) [10.3758/s13428-017-0862-1.](http://dx.doi.org/10.3758/s13428-017-0862-1.)
